# Thermoelectric Properties of *n*-Type Bi_4_O_4_SeX_2_ (X = Cl, Br)

**DOI:** 10.3390/ma16124329

**Published:** 2023-06-12

**Authors:** Tao Wang, Wanghua Hu, Zhefeng Lou, Zhuokai Xu, Xiaohui Yang, Tian Le, Jialu Wang, Xiao Lin

**Affiliations:** 1 Department of Physics, Fudan University, Shanghai 200433, China; 2 Key Laboratory for Quantum Materials of Zhejiang Province, Department of Physics, School of Science, Westlake University, Hangzhou 310030, China; 3 Institute of Natural Sciences, Westlake Institute for Advanced Study, Hangzhou 310024, China; 4 Department of Physics, China Jiliang University, Hangzhou 310018, China

**Keywords:** thermoelectric performance, thermoelectric figure of merit, low thermal conductivity

## Abstract

The multiple anion superlattice Bi_4_O_4_SeCl_2_ has been reported to exhibit extremely low thermal conductivity along the stacking *c*-axis, making it a promising material for thermoelectric applications. In this study, we investigate the thermoelectric properties of Bi_4_O_4_SeX_2_ (X = Cl, Br) polycrystalline ceramics with different electron concentrations by adjusting the stoichiometry. Despite optimizing the electric transport, the thermal conductivity remained ultra-low and approached the Ioffe–Regel limit at high temperatures. Notably, our findings demonstrate that non-stoichiometric tuning is a promising approach for enhancing the thermoelectric performance of Bi_4_O_4_SeX_2_ by refining its electric transport, resulting in a figure of merit of up to 0.16 at 770 K.

## 1. Introduction

Given the fact that more than two-thirds of the world’s produced energy is lost as waste heat in the low and moderate temperature (*T*) range, high-performance thermoelectric (TE) materials have become very important in converting waste heat to electric energy [1,2,3,4,5,6]. TE conversion efficiency is related to a dimensionless TE figure of merit, $ZT=\frac{S^{2}\sigma T}{\kappa_{e}+\kappa_{\mathrm{ph}}}$, where *S* and σ are the Seebeck coefficient and electrical conductivity, respectively, and $\kappa_{e}$ ($\kappa_{\mathrm{ph}}$) represents the electronic (phononic) contribution to thermal conductivity (κ) [7]. According to the formula, ZT could be enhanced by increasing *S* and σ while suppressing κ. However, it is known that the three components S, σ, and $\kappa_{e}$ are closely interconnected, and it seems paradoxical to strengthen one component without satisfying the others’ requirements, given their intimate relationship. In order to improve ZT, several strategies have been developed in the past decades. One is to minimize κ through alloying [8,9,10], nanostructuring [11,12], and phonon engineering [13,14,15,16]. Another is to optimize the TE power factor $PF=S^{2}\sigma$ in intrinsically low κ materials through doping [17,18] and band structure engineering [19,20,21].

Currently, the most widely commercialized semiconductor thermoelectric materials are bipolar Bi_2_Te_3_ alloys [22,23], in which ZT can be up to 1.5 and κ is 2.0–3.5 W m${}^{-1}$ K${}^{-1}$ in low *T* ranges (300–500 K). As homologous compounds of Bi_2_Te_3_, layered bismuth selenium compounds such as Bi_2_Se_3_ [24,25], Bi_2_O_2_Se [17,18,26] (BOS), and BiCuSeO [27,28,29,30] have captured widespread interest in the field of TE research owing to features such as non-toxic, lower-cost elements and excellent chemical and thermal stability in the mid-temperature range (600–900 K). They exhibit high ZT (0.2–1.5) because of their intrinsically low κ (0.8–1.4 W m${}^{-1}$ K${}^{-1}$). Recently, Gibson et al. reported a new van der Waals layered bismuth oxyselenide: a Bi${}_{4}$O${}_{4}$SeCl_2_ (BOSC) superlattice from the mutual intercalating of a van der Waals insulator BiOCl and a quasi-two-dimensional semiconductor of BOS [31]. BOSC exhibits an ultra-low κ (0.10 W m${}^{-1}$ K${}^{-1}$) near room temperature (RT) along the stacking *c*-axis, which is among the lowest of any bulk inorganic material and is only four times the κ of air [32]. Compared with the state-of-the-art Bi_2_Te_3_, BOSC has a much lower κ and electron effective mass, highlighting its potential application in the field of TE. Moreover, Bi${}_{4}$O${}_{4}$SeBr_2_ (BOSB) was reported with κ as low as that of BOSC [33]. Heremans et al. [19] reported the ZT of Bi${}_{4-x}$Sn${}_{x}$O${}_{4}$SeCl_2_ up to 0.033 at 420 K by Sn doping at the Bi site.

**Figure 1:**
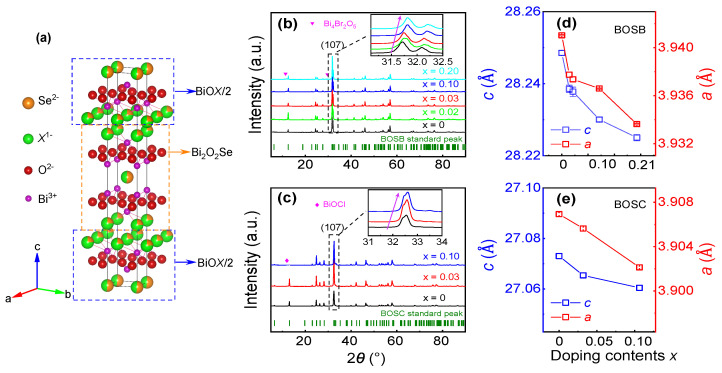
Characterization of Bi${}_{4}$O${}_{4}$Se${}_{1-x}$X${}_{2+x}$ (X = Cl, Br; *x* = 0, 0.02, 0.03, 0.10, 0.20) crystals. (**a**) Layered crystal structure of BOSX consists of alternative stacking of BiOX and BOS layers. Se${}^{2-}$ (orange color) and X${}^{-}$ ions (green color) occupy the same anion site; however, the occupancy is different and has been marked in different volumes. Powder XRD patterns of (**b**) non-stoichiometric tuned BOSB and (**c**) BOSC. The inset is the zoomed-in figure around the (107) reflection, which shows that the peak positions gradually shift to the right as the doping concentration increases. The purple arrow is for eyes to see the trend in the inset subfigure (**b**,**c**). The impurity phase (Bi${}_{4}$Br${}_{2}$O${}_{5}$ or BiOCl) emerges when the doping content *x* is larger than 0.1. The lattice constant as a function of doping content *x* for (**d**) BOSB and (**e**) BOSC.

Here, we report that ZT in Bi${}_{4}$O${}_{4}$SeX_2_ (X = Cl, Br) can be tuned by adjusting the X/Se ratio. X${}^{-}$/Se${}^{2-}$ co-occupy the same site, as shown in Figure 1a. Electrons could be introduced by increasing the X${}^{-}$/Se${}^{2-}$ ratio from the stoichiometry. We prepared several non-stoichiometric BOSC and BOSB ceramic samples with different electron concentrations (*n*) by spark plasma sintering methods. As *n* increases, σ is enhanced, and the ultra-low κ remains, showing the *T*-dependent characteristic of phonon glass. Owing to the ultra-low κ and improved σ, the maximum ZT yielded is 0.167 and 0.161 at 770 K in BOSB and BOSC, respectively.

## 2. Materials and Methods

In order to synthesize Bi${}_{4}$O${}_{4}$Se${}_{1-x}$X${}_{2+x}$ (X = Cl, Br; *x* = 0, 0.02, 0.03, 0.1, 0.2) polycrystalline ceramics, the stoichiometric raw materials of high purity (5N) Se, Bi, Bi_2_O_3_, and BiBr_3_ (BrCl_3_) powders were mixed and pressed into pellets and then heated in an evacuated quartz ampoule at 923 K for one day. After milling, the resulting powders were loaded into a graphite die and sintered using spark plasma sintering (LABOX-650F, Nagaoka, Japan). Each pellet contained approximately 3.2 g of powder, which was packed into a 12 mm inner diameter graphite die set, lined with a rate of 10 min${}^{-1}$, and subjected to 80 MPa of uniaxial pressure. The temperature was monitored using a thermometer. Subsequently, the samples were heated to 823 K at a rate of 50 K min${}^{-1}$, annealed for 20 min, and then cooled to room temperature naturally. During the heating process, the temperature of the sample would typically overshoot by 5–10 K before returning to the set temperature. After cooling to room temperature, the pellets were removed from the die set, and any remaining stain on the surface of the pellets was gently polished away using SiC polishing paper.

The X-ray diffraction (XRD) patterns were performed by using a Bruker D8 advanced X-ray diffractometer with Cu Kα radiation at RT. To satisfy the minimum requirement for Rietveld refinement, the scanning step and collection time were set to approximately 0.01${}^{\circ }$ and 0.1 s, respectively. The accelerating voltage and current were configured to 40 kV and 40 mA, respectively, and the measurement range spanned from 5${}^{\circ }$ to 90${}^{\circ }$. The Rietveld refinement method is based on the least-squares technique, and the sequential order of the entire refinement process is as follows: instrumental factor → scale → background → cell parameters → full width at half maximum of peak → shape parameters → asymmetry parameters → preferred orientation → atomic information. Each step can only be executed once the preceding step has achieved the convergence standard. The micrographs of the sample were acquired using a scanning electron microscope (SEM, Gemini 450, Carl Zeiss AG, Germany), and the composition information derives from Oxford’s energy dispersive X-ray spectrometer (EDS). The carrier concentrations (*n*) were measured in a Quantum Design PPMS-Dynacool equipped with a 9 T magnet. The σ and *S* were measured under He atmosphere from RT to 770 K using a Seebeck Coefficient/Electric Resistance Measuring System (ZEM-3M10, Advance Riko, Japan). The heating rate was set to 50 K min${}^{-1}$. After the temperature stabilized, the Seebeck coefficient and electrical conductivity were measured for the sample at temperature differentials of 2, 5, and 10 K, and the values of σ and *S* were determined by fitting the data at each temperature point. The κ was calculated by the equation $\kappa =DC\rho_{\mathrm{density}}$, where diffusivity *D* and specific heat *C* were measured through the laser flash apparatus (LF467, Netzsch, Germany). The transport property data were collected in 50 K steps within the temperature range of 300 to 773 K, and each temperature was allowed to equilibrate for 5 minutes before measurement. Additionally, five measurements were conducted at each temperature, and their values were averaged to obtain the final value. The mass density $\rho_{\mathrm{density}}$ was obtained by Archimedes method.

The elastic properties of BOSC were calculated by density functional theory (DFT) with the Vienna Ab initio Simulation Package (VASP) and analyzed by VASPKIT tools [34]. The exchange–correlation function was approximated using the Generalized Gradient Approximation (GGA) in the Perdew–Burke–Ernzerhof (PBE) form [35], and the electronic wavefunctions were expanded on a plane-wave basis set with a cutoff energy of 400 eV. The convergence criteria for the residual energy were set to less than 1.0 × 10${}^{-8}$ eV with a force converged to a residual less than 1.0 × 10${}^{-2}$ eV/Å. A 26 × 26 × 4 Gamma centered k-mesh was used for the calculation of elastic constants. The stress–strain method was performed using the VASPKIT package [36].

## 3. Results

As seen in Figure 1a, the unit cell of Bi${}_{4}$O${}_{4}$SeX_2_ (BOSX) is composed of alternating stacking of BiOX and BOS blocks along the *c*-axis, crystallizing in a tetragonal phase with an I4/mmm space group at RT. It is intriguing that Se${}^{2-}$ and X${}^{-}$ share the same occupation when two blocks are mutually intercalated. Figure 1b,c reveal the powder XRD patterns of BOSB and BOSC, respectively. The BOSB patterns exhibit three primary peaks at 31.7${}^{\circ }$, 32.1${}^{\circ }$, and 46.0${}^{\circ }$, which correspond to the (017), (110), and (020) planes, respectively. It should be noted that BOSB and BOSC share virtually identical crystal information, and therefore the difference in peak positions between their diffraction patterns is less than 1${}^{\circ }$. The crystallographic information for both materials has been added to the supplementary materials, with further details available in Tables S1 and S2. No obvious impurity phases were detected at low *n*, and all peaks can be well-indexed and are consistent with Ji’s [33] and Gibson’s results [31]. While in Bi${}_{4}$O${}_{4}$Se${}_{0.8}$Br${}_{2.2}$ and Bi${}_{4}$O${}_{4}$Se${}_{0.9}$Cl${}_{2.1}$ with large *n* and non-stoichiometry, a secondary phase Bi${}_{4}$Br_2_O${}_{5}$ (ICSD #94498) or BiOCl (ICSD #185970) emerges, which means the content of halogen atoms reaches saturation in BOSX. According to the zoomed-in figure around (107) reflection in the inset of Figure 1b,c, the peak gradually shifts towards higher angles as doping content *x* increases, indicating a shrinkage of the unit cell. Accordingly, in Figure 1d,e, the extracted lattice constant decreases monotonically by increasing doping content *x*. The relevant parameters are summarized in Table 1 (see supplementary Figures S1 and S2 for details). This is consistent with the fact that the ionic radii of Cl${}^{-}$ (1.81 Å) and Br${}^{-}$ (1.96 Å) are smaller than that of Se${}^{2-}$ (1.98 Å).

**Figure 2:**
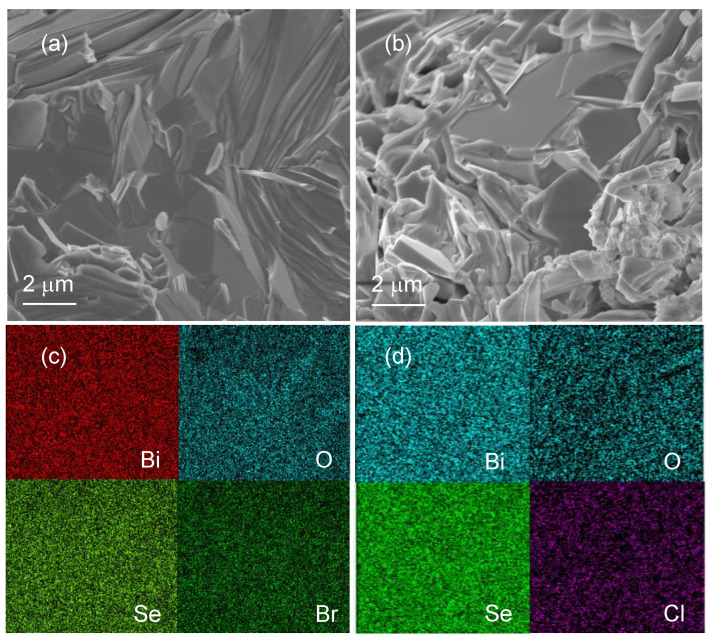
SEM micrographs of (**a**) Bi${}_{4}$O${}_{4}$Se${}_{0.97}$Br${}_{2.03}$ and (**b**) Bi${}_{4}$O${}_{4}$Se${}_{0.97}$Cl${}_{2.03}$ ceramics. EDS mapping analysis (**c**,**d**) from Bi${}_{4}$O${}_{4}$Se${}_{0.97}$Br${}_{2.03}$ and Bi${}_{4}$O${}_{4}$Se${}_{0.97}$Cl${}_{2.03}$ ceramics, respectively.

Figure 2a,b show the the morphology of Bi${}_{4}$O${}_{4}$Se${}_{0.97}$Br${}_{2.03}$ and Bi${}_{4}$O${}_{4}$Se${}_{0.97}$Cl${}_{2.03}$ ceramics, respectively, which exhibit a dense, distinct, layered structure and appear to be free of any noticeable impurities or uneven phases. To analyze the distributions of elements (Bi, O, Se, Br, and Cl), EDS composition mapping was conducted on Bi${}_{4}$O${}_{4}$Se${}_{0.97}$Br${}_{2.03}$ and Bi${}_{4}$O${}_{4}$Se${}_{0.97}$Cl${}_{2.03}$, as depicted in Figure 2c,d. The mapping results show that all the elements abovementioned are uniformly distributed, with no obvious segregation observed.

**Figure 3:**
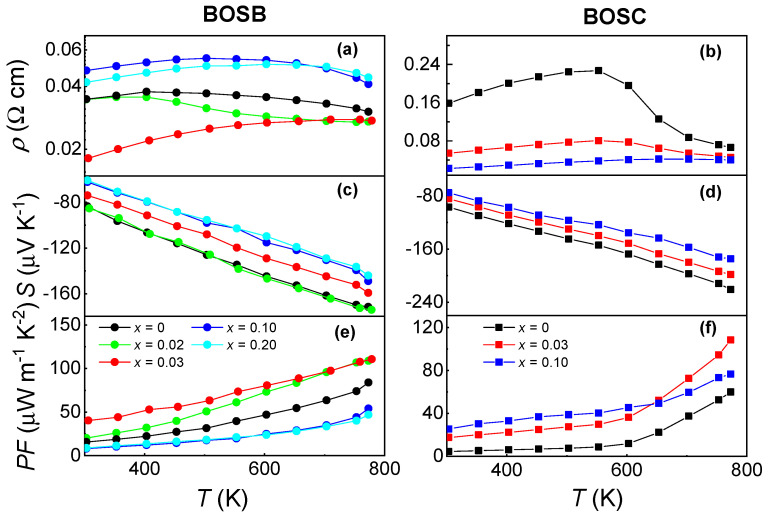
Electrical and thermoelectric transport properties of Bi${}_{4}$O${}_{4}$Se${}_{1-x}$X${}_{2+x}$ (X = Cl, Br; *x* = 0, 0.02, 0.03, 0.10, 0.20) ceramics. *T*-dependent resistivity on a semi-log scale for (**a**) BOSB and (**b**) BOSC. Seebeck coefficients as a function of *T* for (**c**) BOSB and (**d**) BOSC. Power factor of (**e**) BOSB and (**f**) BOSC.

Figure 3a,b show the *T*-dependent resistivity ($\rho =\frac{1}{\sigma }$) for BOSB and BOSC ceramics with various doping contents *x*. The transport parameters are included in Table 1. All samples exhibit metallic behavior near RT, i.e., ρ increases monotonically with increasing *T*. However, at high *T*, the ρ of samples with low doping contents *x* decreases with a rising *T*, indicating the domination of thermally excited carriers in electric transports. This phenomenon is reminiscent of the bipolar effect in TE materials, where an intrinsic excitation causes the conduction band electrons to cross the energy gap, resulting in an increase in the number of charge carriers involved in transport and a decrease in resistivity. However, no evidence of a bipolar effect was observed in the thermal conductivity and thermopower (as shown below). In addition, the large energy gap of BOSC (Δ≈ 1.15 eV) [31] precludes the thermal excitation of electron–hole pairs.

The temperature-dependent *S* of BOSB and BOSC is presented in Figure 3c and Figure 3d, respectively. The *S* decreases with the concentration’s increase, which is consistent with Mott’s equation. The negative sign of *S* indicates electron doping, and a linear *T*-dependence is observed. The average effective mass ($m^{*}$) of BOSB and BOSC at RT, as calculated for samples with high carrier density, is approximately 0.5 $m_{e}$ (see the supplementary information for the details), in line with the first-principles calculation result [31].

*T*-dependent PF of BOSB and BOSC is presented in Figure 3e and Figure 3f, respectively. The PF value of all samples increases monotonically with an increasing *T*. The maximum PF value is up to 108.33 and 112.40 μW m${}^{-1}$ K${}^{-2}$ at 770 K for Bi${}_{4}$O${}_{4}$Se${}_{0.97}$Br${}_{2.03}$ and Bi${}_{4}$O${}_{4}$Se${}_{0.97}$Cl${}_{2.03}$, respectively.

**Figure 4:**
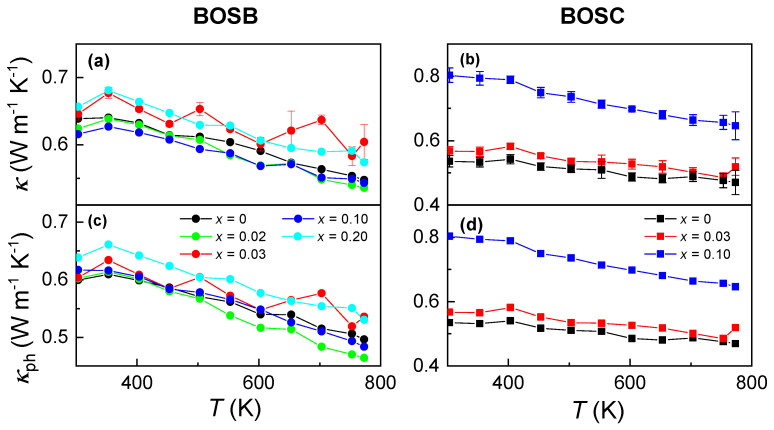
Thermal conductivity of Bi${}_{4}$O${}_{4}$Se${}_{1-x}$X${}_{2+x}$ (X = Cl, Br; *x* = 0, 0.02, 0.03, 0.10, 0.20) ceramics. (**a**,**b**) correspond to the measured κ of BOSB and BOSC, respectively. (**c**,**d**) present $\kappa_{\mathrm{ph}}$ as a function of *T* for BOSB and BOSC, respectively.

Figure 4a,b show κ of BOSX ceramics from RT to 770 K. The evolution of κ with *T* is mild, reminiscent of glass-like behavior. It is known that κ is composed of contributions from phonons and electrons: $\kappa =\kappa_{\mathrm{ph}}+\kappa_{e}$. By using $\kappa_{e}$ estimated from the Wiedemann–Franz law ($\kappa_{e}=LT/\rho$ and *L* is the Lorentz number), $\kappa_{\mathrm{ph}}$ is yielded as seen in Figure 4c,d. The Lorentz numbers presented in Figure S3 of the supplementary information are determined by fitting the experimental *S* values to an estimate of the reduced chemical potential (η). The ratio of $\kappa_{\mathrm{ph}}/\kappa$ amounts to 97% even in highly conductive samples, demonstrating the dominant role of phonons in the heat flow. Moreover, Bi${}_{4}$O${}_{4}$Se${}_{0.90}$Cl${}_{2.1}$ exhibits a higher thermal conductivity compared to the other two components due to the presence of impurity phase BOCl, which possesses higher thermal conductivity.

In order to gain a deep understanding of the ultra-low $\kappa_{\mathrm{ph}}$, we calculated the average ($v_{a}$) sound velocity for BOSC using density functional theory calculations ($v_{a}\approx$ 2010 m s${}^{-1}$; see details in the supplementary material). A rough estimation of the phonon mean free path ($\ell_{\mathrm{ph}}$) can be made through $\kappa_{\mathrm{ph}}=\frac{1}{3}C_{v}\nu {}_{a}\ell_{\mathrm{ph}}$, where $C_{v}$ ($C_{v}$ = $C\rho_{\mathrm{density}}$) represents the specific heat capacity per unit volume. At 770 K, the $\ell_{\mathrm{ph}}$ of Bi${}_{4}$O${}_{4}$Se${}_{0.97}$Cl${}_{2.03}$ is approximately 4.65 Å, which is slightly larger than the in-plane lattice constant (*a* = 3.906 Å). This indicates that the system is approaching the Ioffe—Regel limit ($\ell_{\mathrm{ph}}=a$).

Below, we discuss the origin of ultra-low $\kappa_{\mathrm{ph}}$. The origin can be explained by two factors; first of all, the lattice mismatch at the bridging interface between BiOCl (BiOBr) and Bi_2_O_2_Se is large—about 3.1% (1.3%)—and distorts the in-plane lattice vibration. The in-plane structure instability will soften the anharmonic transverse acoustic (TA) phonon and reduce the velocity of out-plane TA phonons [32,37]. Second, phonon scattering could be strengthened by lattice defects from the X/Se co-occupation [31].

**Figure 5:**
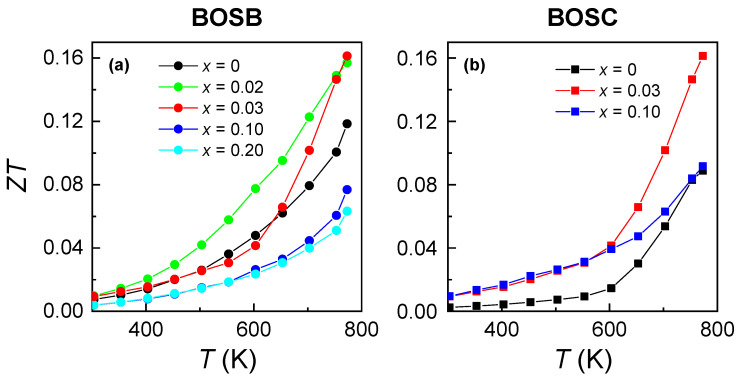
Thermoelectric figure of merit as a function of *T* for (**a**) Bi${}_{4}$O${}_{4}$Se${}_{1-x}$Br${}_{2+x}$ and (**b**) Bi${}_{4}$O${}_{4}$Se${}_{1-x}$Cl${}_{2+x}$ (*x* = 0, 0.02, 0.03, 0.10, 0.20).

Figure 5a,b present the *T*-dependent ZT for BOSB and BOSC, respectively. At 770 K, the maximum ZT values of 0.167 and 0.161 were obtained for Bi${}_{4}$O${}_{4}$Se${}_{0.97}$Br${}_{2.02}$ and Bi${}_{4}$O${}_{4}$Se${}_{0.97}$Cl${}_{2.03}$, which are the highest values compared with that of BOSC [19,33,38], signalling *n*-type BOSB and BOSC polycrystals may have a promising future in the medium-*T* TE field.

While the TE performance of BOSX may not be impressive, there are various strategies that can improve its ZT. For instance, directional sintering technology can be employed to form texture along the [001] crystallographic direction [32], thereby reducing the $\kappa_{\mathrm{ph}}$ of BOSC and BOSB. Refining nanosheets through shear exfoliation [39] or mixing highly conductive materials like graphite nanosheets [40] are feasible scenarios for enhancing the electrical conductance of layered materials. The density of states near the Fermi level can be increased through doping BOSC and BOSB at Bi or O sites, based on resonant level theory [19], which can help optimize *S*. Moreover, doping superparamagnetic nanoparticles [41] like Fe, Co, and Ni can simultaneously improve the *S* and σ of BOSC and BOSB within a reasonable range. Given the weak van der Waals interlayer interaction of BOSX, reducing the crystal dimensionality to two dimensions may prove to be advantageous for the production and application of thermoelectric devices within its low-temperature range.

## 4. Conclusions

In summary, we have demonstrated that BOSX polycrystalline ceramics, composed of BiOX and BOS blocks, have the potential to become effective TE materials owing to their ultra-low thermal conductivity. Electrons are introduced by adjusting the stoichiometry of the X/Se ratios. The maximum ZT values are as high as 0.167 and 0.161 at 770 K for BOSB and BOSC, respectively. Our study highlights the importance of non-stoichiometric tuning in optimizing the TE properties of materials and provides new insights into the relationship between electron concentration, thermal conductivity, and electric transport in complex anion superlattice materials. Moreover, we have proposed several strategies, including doping and sample preparation, to enhance the TE performance of this material. As a layered van der Waals material, reducing the dimensionality of this material and fabricating it into TE devices will be the future direction of research and application of this system.

## Figures and Tables

**Table 1:** Transport parameters for BOSX samples. $n_{300K}$ and $\rho_{300K}$ are the Hall *n* and the ρ measured at 300 K, respectively. *c* and *a* are refined lattice constants of BOSC and BOSB. $ZT_{770K}$ is the ZT at 770 K.

Samples	Label	$n_{300K}\left(10^{18}\;{\mathrm{cm}}^{-3}\right)$	$\rho_{300K}\;\left(\Omega \;\mathrm{cm}\right)$	*c* (Å)	*a* (Å)	${\mathrm{ZT}}_{770K}$
Bi${}_{4}$O${}_{4}$SeBr${}_{2}$	*x* = 0	46	0.035	28.249	3.941	0.118
Bi${}_{4}$O${}_{4}$Se${}_{0.98}$Br${}_{2.02}$	*x* = 0.02	48	0.035	28.239	3.938	0.167
Bi${}_{4}$O${}_{4}$Se${}_{0.97}$Br${}_{2.03}$	*x* = 0.03	50	0.025	28.237	3.937	0.157
Bi${}_{4}$O${}_{4}$Se${}_{0.9}$Br${}_{2.1}$	*x* = 0.10	59	0.048	28.230	3.936	0.077
Bi${}_{4}$O${}_{4}$Se${}_{0.8}$Br${}_{2.2}$	*x* = 0.20	64	0.042	28.225	3.934	0.063
Bi${}_{4}$O${}_{4}$SeCl${}_{2}$	*x* = 0	0.45	0.181	27.073	3.907	0.089
Bi${}_{4}$O${}_{4}$Se${}_{0.97}$Cl${}_{2.03}$	*x* = 0.03	3.2	0.054	27.065	3.906	0.161
Bi${}_{4}$O${}_{4}$Se${}_{0.90}$Cl${}_{2.1}$	*x* = 0.10	14	0.022	27.059	3.902	0.092

## Data Availability

The data that support the findings of this study are available within the article and its supplementary material.

## Supplementary Materials

The supplementary material includes hall resistivity, refined XRD patterns and the DFT calculations results of BOSX mechanical properties. It can be downloaded at: https://www.mdpi.com/article/10.3390/ma16124329/s1, and Figure S1: Hall resistivity at 300 K for BOSB and BOSB ( *x* = 0, 0.02, 0.03, 0.10, 0.20); Figure S2: XRD refinement patterns of BOSX (X = Cl, Br; *x* = 0, 0.02, 0.03, 0.10, 0.20). Figure S3: The temperature-dependent the Lorentz number for BOSC and BOSB (*x* = 0, 0.02, 0.03, 0.10, 0.20); Table S1: BOSB crystalline information; Table S2: BOSC crystalline information. Refs. [42,43,44] are cited in the supplementary materials.
