# Supplementary material for: Thermoelectric Properties of n-Type Bi4O4SeX2 (X = Cl, Br)
Source: Materials (Basel). 2023 Jun 12;16(12):4329. doi: 10.3390/ma16124329 (PMC10303193; doi:10.3390/ma16124329)
Supplement: Supplementary file 1 [file materials-16-04329-s001.zip › materials-2398152-supplementary.pdf]

# Supplementary information for "Thermoelectric properties of $n$-type $Bi_4O_4SeX_2$ (X = Cl, Br)"

Tao Wang,[1,2,3] Wanghua Hu,[1,2,3] Zhefeng Lou,[2,3] Zhuokai Xu,[2,3] Xiaohui Yang,[2,3,4] Tian Le,[2,3] Jialu Wang,[2,3] and Xiao Lin[2,3]

[1]*Department of Physics, Fudan University, Shanghai 200433, China*

[2]*Key Laboratory for Quantum Materials of Zhejiang Province, Department of Physics, School of Science, Westlake University, Hangzhou 310030, China*

[3]*Institute of Natural Sciences, Westlake Institute for Advanced Study, Hangzhou 310024, China*

[4]*Department of Physics, China Jiliang University, Hangzhou 310018, Zhejiang, China*

## I. HALL RESISTIVITY

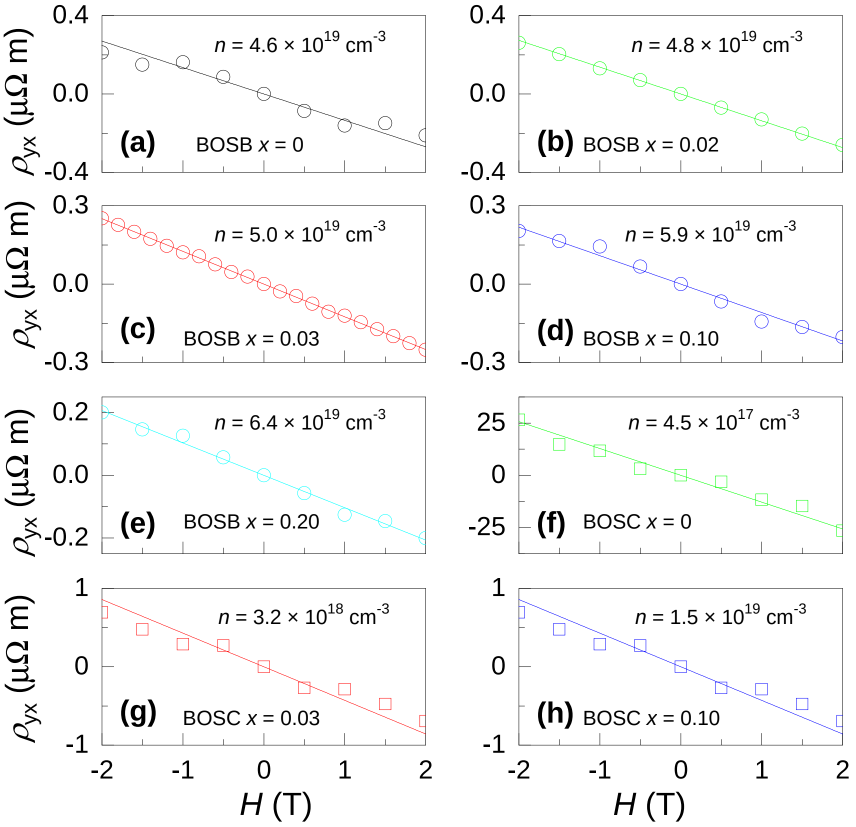


**Figure S1.** Hall resistivity at 300 K for BOSB and BOSB ( $x$ = 0, 0.02, 0.03, 0.10, 0.20).

Fig. S1 presents the Hall resistivity ($\rho_{\rm yx}$) for all samples at room-temperature. The negative slope ($\rho_{\rm yx}/B$) demonstrates that electrons dominate the electrical transport. The carrier concentration ($n$) is estimated by a single-band model $n = \frac{1}{R_{\rm H}e}$, where $R_{\rm H}$ is the hall coefficient.

## II. XRD REFINEMENT PATTERNS

Fig. S2 shows the Rietveld refinement XRD patterns of ten samples, the refinement software is FullProf Suite, XRD peak shape is fitting by Pseudo-Voigt function, the refinement results ($R_{\rm p}$, $R_{\rm wp}$) have been achieved by least-squares technique and shown in each figure.

Table S1 and Table S2 show the BOSX crystalline information, due to same space group ($I4/mmm$) and similar atomic occupancy as well lattice constants (See table 1 of main text), there is only a slight difference in the angle of the peak position for BOSB and BOSC.

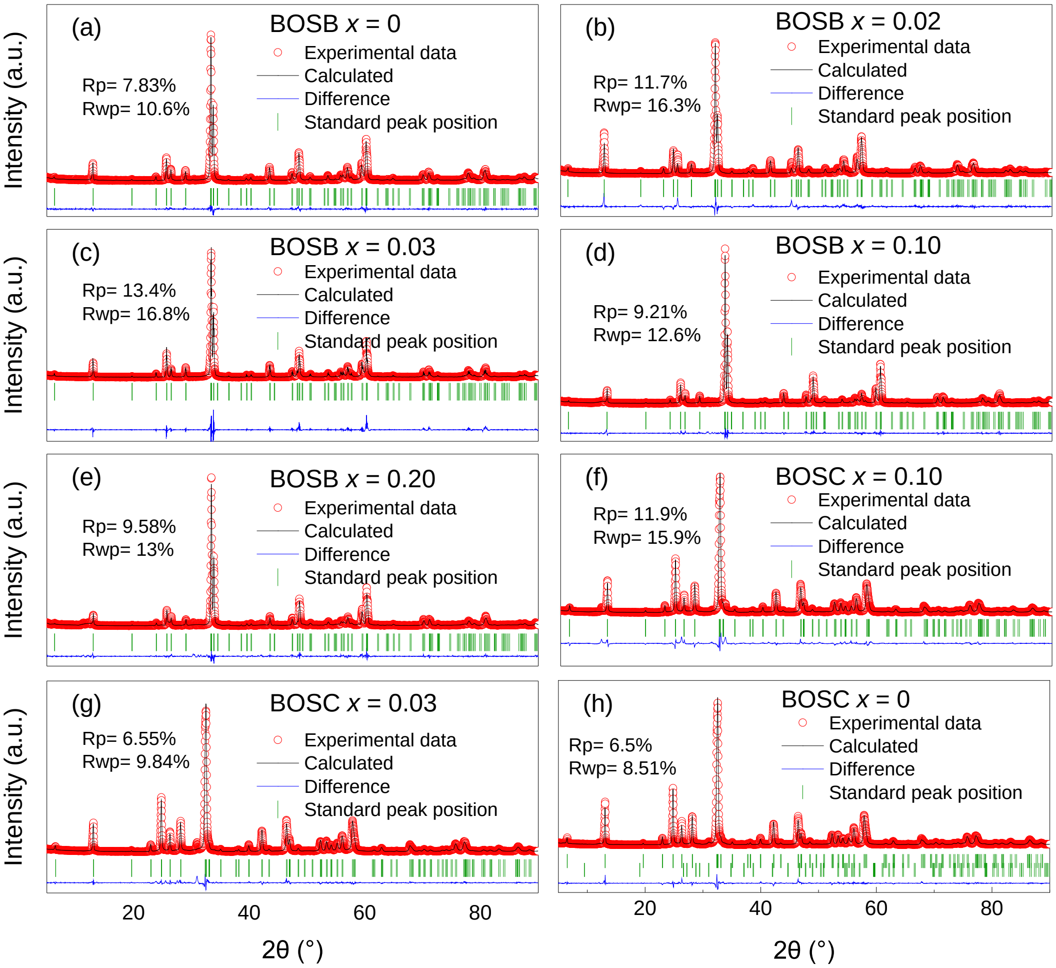


**Figure S2.** XRD refinement patterns of BOSX (X = Cl, Br; $x = 0, 0.02, 0.03, 0.10, 0.20$).

**Table S1.** BOSB crystalline information

| Number | Element | Label | x | y | z | Occ. | U | Site | Sym |
|---|---|---|---|---|---|---|---|---|---|
| 1 | Bi | Bi1 | 1.00000 | 1.00000 | 0.56827 | 1.00000 | 0.00700 | 4e | 4mm |
| 2 | Bi | Bi2 | 0.50000 | 0.50000 | 0.66148 | 1.00000 | 0.01300 | 4e | 4mm |
| 3 | Se | Se1 | 0.50000 | 0.50000 | 0.50000 | 0.52000 | 0.00800 | 2a | 4/mmm |
| 4 | Se | Se2 | 1.00000 | 1.00000 | 0.70961 | 0.24200 | 0.00900 | 4e | 4mm |
| 5 | O | O1 | 0.50000 | 1.00000 | 0.61190 | 1.00000 | 0.00800 | 8g | 2mm |
| 6 | Br | Br1 | 0.50000 | 0.50000 | 0.50000 | 0.48000 | 0.00800 | 2a | 4/mmm |
| 7 | Br | Br2 | 1.00000 | 1.00000 | 0.70961 | 0.75800 | 0.00900 | 4e | 4mm |

Space group $I4/mmm$, BOSB lattice constants $a = b = 3.93007$ Å

$c = 28.1763$ Å, $V = 435.196$ Å$^3$, $\rho_{density} = 8.6899$ g/cm$^3$

**Table S2.** BOSC crystalline information

| Number | Element | Label | x | y | z | Occ. | U | Site | Sym |
|---|---|---|---|---|---|---|---|---|---|
| 1 | Bi | Bi1 | 1.00000 | 1.00000 | 0.56819 | 1.00000 | 0.007 | 4e | 4mm |
| 2 | Bi | Bi2 | 0.50000 | 0.50000 | 0.65656 | 1.00000 | 0.01300 | 4e | 4mm |
| 3 | Se | Se1 | 0.50000 | 0.50000 | 0.50000 | 0.59000 | 0.00800 | 2a | 4/mmm |
| 4 | Se | Se2 | 1.00000 | 1.00000 | 0.70662 | 0.21000 | 0.00900 | 4e | 4mm |
| 5 | O | O1 | 0.50000 | 1.00000 | 0.60895 | 1.00000 | 0.00800 | 8g | 2mm |
| 6 | Cl | Cl1 | 0.50000 | 0.50000 | 0.50000 | 0.41000 | 0.00800 | 2a | 4/mmm |
| 7 | Cl | Cl2 | 1.00000 | 1.00000 | 0.70662 | 0.79000 | 0.00900 | 4e | 4mm |

Space group $I4/mmm$, BOSC lattice constants $a = b = 3.89373$ Å

$c = 27.02904$ Å, $V = 410.84$ Å$^3$, $\rho_{density} = 8.4862$ g/cm$^3$

## III. CALCULATION OF EFFECTIVE MASS AND LORENZ NUMBER

In the context of the single parabolic band assumption, the effective mass ($m^*$) and Lorenz number ($L$) can be determined by the following expressions:

$$m^* = \frac{h^2}{2k_{\mathrm{B}}T}\left[\frac{n}{4\pi F_{1/2}(\eta)}\right]^{2/3} \tag{1}$$

$$S = \pm\frac{k_{\mathrm{B}}}{e}\left[\frac{(r+5/2)\,F_{r+3/2}(\eta)}{(r+3/2)\,F_{r+1/2}(\eta)} - \eta\right] \tag{2}$$

$$F_n(\eta) = \int_0^{\infty}\frac{\chi^n}{1+\exp(\chi-\eta)}\mathrm{d}\chi \tag{3}$$

$$L = \left(\frac{k_{\mathrm{B}}}{e}\right)^2\left\{\frac{(r+7/2)F_{5/2+r}(\eta)}{(r+3/2)F_{1/2+r}(\eta)} - \left[\frac{(r+5/2)F_{3/2+r}(\eta)}{(r+3/2)F_{1/2+r}(\eta)}\right]^2\right\} \tag{4}$$

where $\eta$ denotes the reduced Fermi level, which is calculated as the ratio of Fermi energy $E_{\mathrm{F}}$ to $k_{\mathrm{B}}T$ (the Boltzmann constant multiplied by the temperature in Kelvin). The remaining physical parameters used in the equations include h (the Planck constant), $F_n$ (the nth order Fermi integral), $\chi$ (reduced energy, defined as $\chi = E/k_{\mathrm{B}}T$) and $e$ (the elementary charge). Additionally, the scattering exponent $r = -1/2$, where Acoustic Phonon Scattering (APS) is the dominant carrier scattering mechanisms in the samples.

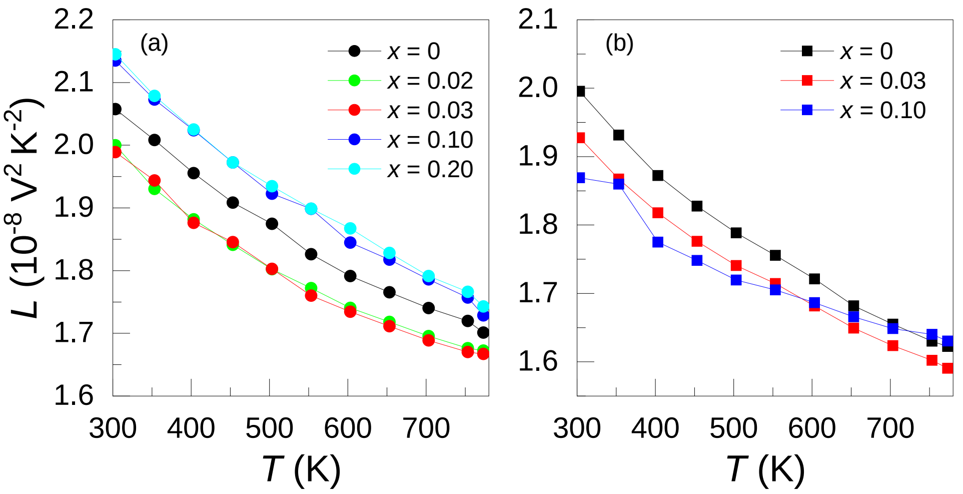


**Figure S3.** The temperature-dependent the Lorentz number for BOSC and BOSB ($x = 0$, 0.02, 0.03, 0.10, 0.20).

## IV. ELASTIC CONSTANTS

For BOSC with $I4/mmm$ space group, there are six independent elastic constants $C_{11}$, $C_{12}$, $C_{13}$, $C_{33}$, $C_{44}$ and $C_{66}$ corresponding to 144.83, 71.69, 39.83, 63.72, 21.16 and 58.89 GPa. The elastic stability criteria for tetragonal crystal system are shown as follows[1]:

$$C_{11} > |C_{12}| \tag{5}$$

$$2 * C_{13}^2 < C_{33} * (C_{11} + C_{12}) \tag{6}$$

$$C_{44} > 0 \tag{7}$$

$$C_{66} > 0 \tag{8}$$

Average mechanical properties[2] of bulk BOSC polycrystal include bulk modulus ($B$) 57.54 GPa, shear modulus ($G$) 27.77 GPa, longitudinal velocity ($v_\mathrm{l}$), transverse sound velocity ($v_\mathrm{t}$) and average sound velocity ($v_\mathrm{a}$) [3], which can be calculated by formula (9)-(13).

$$B = (2C_{11} + C_{33} + 2C_{12} + 4C_{13})/9 \tag{9}$$

$$G = (2C_{11} + C_{33} - C_{12} - 2C_{13} + 6C_{44} + 3C_{66})/15 \tag{10}$$

$$v_\mathrm{l} = \sqrt{\frac{(B + (4/3)G)}{\rho_\mathrm{density}}} \tag{11}$$

$$v_\mathrm{t} = \sqrt{\frac{G}{\rho_\mathrm{density}}} \tag{12}$$

$$v_{\mathrm{a}} = \left[\frac{1}{3}\left(\frac{1}{v_{\mathrm{l}}^3} + \frac{2}{v_{\mathrm{t}}^3}\right)\right]^{-\frac{1}{3}} \tag{13}$$

## References


[1] F. Mouhat and X. Coudert, François, Phys. Rev. B **90**, 224104 (2014).

[2] L. Liu, D. Wang, Y. Zhong, and C. Hu, Appl. Sci. **9**, 1267 (2019).

[3] Y. Duan, Y. Sun, M. Peng, and S. Zhou, J. Alloys Compd. **595**, 14 (2014).
